# Interpregnancy interval and risk of recurrence following tubal ectopic pregnancy: retrospective cohort study from UK tertiary center

**DOI:** 10.1002/uog.29262

**Published:** 2025-06-05

**Authors:** W. M. Dooley, J. Farren, L. V. De Braud, S. A. Solangon, N. Thanatsis, B. H. Al Wattar, D. Jurkovic

**Affiliations:** ^1^ EGA Institute for Women's Health, Faculty of Population Health Sciences University College London (UCL) London UK; ^2^ Beginning Assisted Conception Unit Epsom and St Helier University Hospitals London UK

**Keywords:** ectopic pregnancy, extrauterine pregnancy, pregnancy complications, tubal pregnancy

## Abstract

**Objective:**

To assess the effect of interpregnancy interval on the odds of recurrence of tubal ectopic pregnancy (TEP) following expectant or surgical management.

**Methods:**

This was a retrospective cohort study conducted at a tertiary early pregnancy unit (EPU) in London, UK. Patients diagnosed with TEP following spontaneous conception, who had expectant or surgical management and who attended the EPU between December 2008 and January 2021 were included. Univariate and multivariate regression analyses were conducted to explore the association between the odds of recurrence of ectopic pregnancy and various factors, including maternal history, interpregnancy interval and management method of the index TEP, and analyses were adjusted for confounders. The main outcome measure was the odds of recurrence of extrauterine ectopic pregnancy in women presenting with a subsequent pregnancy.

**Results:**

A total of 1386 women with TEP were included, of whom 626 (45.2%) presented with a subsequent pregnancy. Fifty‐nine of these women were excluded, as their subsequent pregnancy was conceived via *in‐vitro* fertilization. From the remaining 567 women, 59 (10.4%) were diagnosed with recurrent extrauterine ectopic pregnancy. An interpregnancy interval of 6–18 months was associated with four times the odds of recurrence compared with an interval of ≤ 3 months (odds ratio (OR), 4.05 (95% CI, 1.37–12.03)). Women with two or more previous TEPs had more than three times the odds of recurrence compared to those with one previous TEP (OR, 3.27 (95% CI, 1.13–9.42)). Surgical management of the index TEP was associated with similar odds of recurrence as expectant management (OR, 1.26 (95% CI, 0.72–2.20)).

**Conclusions:**

Rapid conception after TEP is associated with low odds of recurrence. Therefore, purposeful delay to conception after TEP, including those managed expectantly, should not be recommended. Women with conception delay or a history of more than one ectopic pregnancy are at high risk of recurrent extrauterine ectopic pregnancy. © 2025 The Author(s). *Ultrasound in Obstetrics & Gynecology* published by John Wiley & Sons Ltd on behalf of International Society of Ultrasound in Obstetrics and Gynecology.

## INTRODUCTION

Tubal ectopic pregnancy (TEP) affects approximately 35 000 women in the UK annually, with a rate of 11 per 1000 pregnancies and a documented maternal mortality of 0.2 per 1000 estimated cases of ectopic pregnancy^1^, ^2^. The reported rate of normally sited (eutopic) pregnancy following TEP is 50–70%^3^. Women with a history of TEP have been shown to have a five‐fold increased risk of recurrent TEP^4^, with reported recurrence rates of 10–19%^5^, ^6^. Risk factors for recurrent TEP include nulliparity, history of pelvic surgery, subfertility and pelvic inflammatory disease^6^, ^7^, ^8^, ^9^. One study investigating 125 women following medical management of TEP found no difference in recurrence risk based on interpregnancy interval^10^. However, there have been no studies assessing the impact of interpregnancy interval after expectant or surgical management.

In 2007, the World Health Organization recommended waiting at least 6 months after experiencing miscarriage before trying to conceive again^11^. However, since a more recent meta‐analysis concluded that an interpregnancy interval of less than 6 months was associated with a lower risk of miscarriage, women have been supported in trying to conceive as soon as they feel ready^12^. There is no equivalent guidance on interpregnancy interval after a previous ectopic pregnancy, save for a recommendation to avoid conception within 3 months of methotrexate administration owing to the risk of teratogenicity^2^.

Expectant management of TEP is increasingly prevalent, but there is a potential concern that slow physical resolution within the Fallopian tube could increase the risk of recurrent extrauterine ectopic pregnancy. A previous study of our group demonstrated that two‐thirds of TEP cases are not visible on ultrasound 2 weeks after normalization of human chorionic gonadotropin (hCG) levels, and 95% of cases are not visible after 78 days^13^. However, it would be prudent not to extrapolate this result to imply a rapid restoration of tubal function; instead, investigation should focus on whether quicker conception may in fact be associated with a higher risk of recurrence. In contrast, purposeful delay may coincide with a natural decline in fertility, resulting in potential harm from an unnecessarily cautious approach^10^.

The primary objective of this study was to assess the effect of interpregnancy interval on the odds of recurrence of extrauterine ectopic pregnancy following expectant or surgical management. Additionally, we assessed the odds of recurrence based on the method of TEP management and other potential risk factors.

## METHODS

### Study design and participants

We conducted a retrospective cohort study using routinely collected data stored in a bespoke clinical database. We included all women diagnosed with TEP at our tertiary center between December 2008 and January 2021 who underwent expectant or surgical management. We excluded women who were managed medically as they were routinely advised to delay their next pregnancy for 3 months after methotrexate administration. Women who conceived after *in‐vitro* fertilization (IVF) in their index or subsequent pregnancy were also excluded. Such conceptions bypass the requirement for tubal patency and, as such, have the potential to obscure understanding of the residual natural reproductive function. We were advised by the Joint Research Office of University College London and University College London Hospitals, London, UK, that no formal ethical approval was required for this study, as all data were collected as part of routine care and anonymized before analysis.

### Setting

The study was conducted in a tertiary early pregnancy unit (EPU) at University College London Hospitals, London, UK. Our unit has an accessible walk‐in service for women with early pregnancy issues or previous pregnancy loss, and we recommend that all women with a previous ectopic pregnancy attend at 5 weeks' gestation in subsequent pregnancies. We have a standardized approach to the diagnosis and management of TEP, as described in our recent publication^14^. All women who attend our unit are reviewed by either a consultant gynecologist with expertise in early pregnancy, or a clinical fellow working under their supervision. Women are assessed clinically before undergoing a transvaginal ultrasound scan using high‐end equipment (Voluson E8; GE Healthcare, Zipf, Austria).

### Exposure

In all cases, the diagnosis of TEP was made by direct visualization of an extrauterine pregnancy on ultrasound, with typical structure, rather than based on biochemical markers alone.

The morphology of the TEP was classified as one of the following: (1) gestational sac containing an embryo, with visible cardiac activity; (2) gestational sac containing an embryo, with no visible cardiac activity; (3) gestational sac containing a yolk sac, with no visible embryo; (4) empty gestational sac, with no visible additional structures; or (5) solid swelling. Interstitial pregnancies were categorized as tubal, in accordance with the European Society of Human Reproduction and Embryology classification^15^.

Successful expectant management was defined as a decline in the level of serum hCG to < 20 IU/L without the need for any additional medical or surgical treatment. Surgical management of TEP was recommended to those presenting with moderate or severe pain, and to those who were found to have significant hemoperitoneum on ultrasound examination, defined by the presence of blood clots within the lesser pelvis or hemoperitoneum extending above the fundus of the uterus^16^. Surgical management was also recommended if the TEP was found to contain a live embryo, if the trophoblastic tissue had a mean diameter of > 3 cm or if the serum hCG level at diagnosis was > 1500 IU/L. More than half of the women were recommended to initially have expectant management. Over the study period, medical management of TEP was uncommon in our unit. It was offered after consultant review in specific circumstances, mainly in the context of clinical trials. During this study, two clinical trials took place: GEM3 (comparing gefitinib and methotrexate *vs* methotrexate alone for TEP) and a further study comparing methotrexate with expectant management for TEP^17^, ^18^. Women recruited to these trials were excluded from the present study.

Surgical treatment was recommended if hCG levels were increasing persistently at follow‐up appointments during expectant or medical management, or if women developed increasing abdominal pain. We classified surgery as ‘radical’ when a salpingectomy was performed or ‘conservative’ when the Fallopian tube was not removed. Conservative surgery included a salpingotomy or removal of the trophoblastic tissue partially protruding through the fimbrial ostium, in cases of failing TEPs. The final management method was documented for each woman.

For all women who had a TEP during the study period (henceforth referred to as the ‘index’ TEP), their demographic characteristics and details of any previous pregnancies were recorded.

Prior pregnancy losses not visualized on ultrasound (either a biochemical pregnancy for which an ultrasound was not performed or a resolved pregnancy of unknown location (PUL) on ultrasound) were assumed to be correctly sited for the purposes of the analysis.

Following successful expectant or surgical management, the women were informed that they could try to conceive again without delay.

If a woman presented with a subsequent pregnancy, we calculated the interpregnancy interval as the time between when the index TEP was diagnosed and the first day of the last menstrual period (LMP) of their subsequent pregnancy; or, if the LMP date was not available, the expected LMP date based on the initial ultrasound scan was used. The location of the pregnancy, along with any treatment required if it was ectopic, was recorded.

### Data collection and quality assurance

Cases were identified using the clinical database (PIA Fetal Database, version 2.23; Viewpoint Bildverarbeitung GmbH, Munich, Germany), into which all data were recorded prospectively. We assessed the outcome of the subsequent pregnancy following the index TEP, to assess the odds of recurrence. The study was registered with the Research Registry (reference number: 9921).

### Statistical analysis

Statistical analysis of the data was performed using SPSS for Windows version 25 (IBM Corp., Armonk, NY, USA). Categorical variables were summarized as *n* (%) of patients in each category. Continuous variables were expressed as mean ± SD, if found to be approximately normally distributed, or as median (interquartile range (IQR)) if not. *P* < 0.05 was used to indicated statistical significance.

The outcome of interest was whether women with a subsequent pregnancy had an ectopic pregnancy; this was considered as a binary outcome (yes/no), and the analysis was performed using logistic regression. The first stage of the analysis considered the separate association between each potential risk factor and the outcome, in a series of univariable analyses. Subsequently, the joint association between the factors and subsequent TEP was examined using multivariable analysis. To restrict the number of variables in this second stage, only those showing any association with the outcome from the univariable analyses (*P* < 0.1) were included. A backwards selection procedure was used to retain only the significant variables in the final model.

## RESULTS

### Index TEP

**Figure 1:**
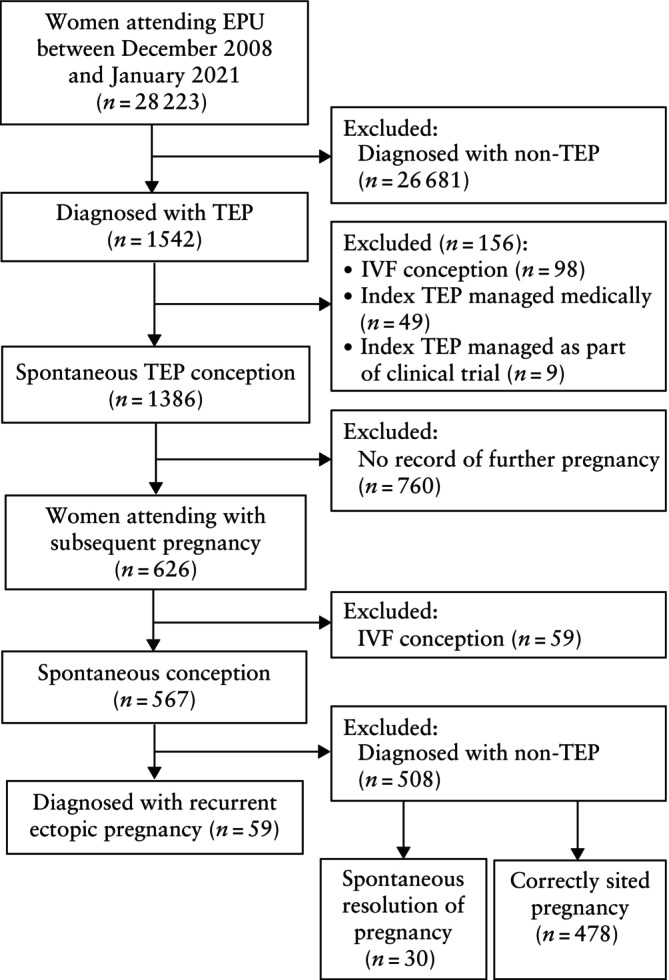
Flowchart of women with tubal ectopic pregnancy (TEP) included in study, including those with subsequent spontaneous pregnancy and recurrent extrauterine ectopic pregnancy. EPU, early pregnancy unit; IVF, *in‐vitro* fertilization.

In total, 28 223 pregnant women attended the EPU for assessment during the study period, of whom 1542 (5.5%) were diagnosed with a TEP. We excluded 98 women who conceived via IVF, 49 who were managed medically and nine who were managed as part of blinded clinical trials. Therefore, a total of 1386 women with a TEP were included in this study (Figure 1). The median gestational age at the time of diagnosis of the index TEP was 6 + 3 (IQR, 5 + 5 to 7 + 4) weeks.

The demographic characteristics of the women included in the study are shown in Table S1. Fifty‐nine (4.3%) women had a history of previous TEP prior to the index TEP. Out of all the women included, 739 (53.3%) had initial expectant management, which was successful in 504/739 (68.2%) women. Surgery was conducted as the initial management strategy in 647/1386 (46.7%) women; however, 882/1386 (63.6%) women in total ultimately required surgical management. The morphological features on ultrasound examination and the management method of the index TEP are shown in Table S2.

### Subsequent pregnancy

During the study period, 626/1386 (45.2%) women attended with a subsequent pregnancy following the index TEP, of which 59 were conceived via IVF and were excluded from the analysis. Of the remaining 567 women with a subsequent pregnancy, 59 (10.4%) were found to have a recurrent extrauterine ectopic pregnancy.

There were 58 TEPs and one ovarian ectopic pregnancy. Out of the 58 TEPs, 37 (63.8%) were on the contralateral side to the index TEP and 21 (36.2%) were ipsilateral. Of the 59 women with recurrent extrauterine ectopic pregnancy, 23 (39.0%) had initial surgical management and 36 (61.0%) had initial expectant management, which was successful in 27/36 (75.0%) cases. One of the nine women with failed expectant management was then managed medically, and eight women ultimately had surgical intervention. Overall, 31/59 (52.5%) women had surgical management, which represented removal of both Fallopian tubes for 12 (38.7%) of these women. The morphology on ultrasound examination and the management method of these cases of recurrent extrauterine ectopic pregnancy are shown in Table S3.

### Factors associated with recurrent ectopic pregnancy

Univariate analysis of factors associated with recurrence of ectopic pregnancy, including demographic factors, past pregnancy history, interpregnancy interval and management method of the index TEP, is shown in Table 1. An interpregnancy interval of 6–18 months (odds ratio (OR), 4.05 (95% CI, 1.37–12.03)) and > 18 months (OR, 5.84 (95% CI, 1.91–17.82)) were associated with significantly higher odds of recurrence compared with an interval of ≤ 3 months.

**Table 1:** Univariate analysis of factors associated with odds of recurrent extrauterine ectopic pregnancy (*n* = 567)

Variable	Subsequent ectopic pregnancy	OR (95% CI)	*P*
Maternal age at index TEP*	—	0.85 (0.65–1.11)	0.24
Gravidity at index TEP			
1	26/203 (12.8)	Reference	0.16
≥ 2	33/364 (9.1)	0.68 (0.39–1.17)	
Parity at index TEP			
0	40/339 (11.8)	Reference	0.19
≥ 1	19/228 (8.3)	0.68 (0.38–1.21)	
Previous miscarriage†			
No	38/401 (9.5)	Reference	0.26
Yes	21/166 (12.7)	1.38 (0.79–2.44)	
Previous TOP†			
No	52/444 (11.7)	Reference	0.06
Yes	7/123 (5.7)	0.45 (0.20–1.03)	
Previous TEP†			
No	54/548 (9.9)	Reference	0.03
Yes	5/19 (26.3)	3.27 (1.13–9.42)	
Previous Cesarean section†			
0	51/487 (10.5)	Reference	0.90
≥ 1	8/80 (10.0)	0.95 (0.43–2.08)	
IUCD *in situ*			
No	58/561 (10.3)	Reference	0.62
Yes	1/6 (16.7)	1.73 (0.20–15.1)	
Gestational age at presentation‡			
< 40 days	17/151 (11.3)	Reference	0.92
41–55 days	30/269 (11.2)	0.99 (0.53–1.86)	
≥ 56 days	8/83 (9.6)	0.84 (0.35–2.04)	
Morphology of index TEP			
Solid swelling	22/258 (8.5)	Reference	0.31
GS	24/208 (11.5)	1.40 (0.76–2.57)	
GS + YS	3/44 (6.8)	0.78 (0.22–2.74)	
Embryo	2/14 (14.3)	1.79 (0.37–8.50)	
Embryo + CA	8/43 (18.6)	2.45 (1.01–5.93)	
Initial management of index TEP			
Expectant	31/350 (8.9)	Reference	0.13
Surgical	28/217 (12.9)	1.52 (0.89–2.62)	
Final management of index TEP			
Expectant	22/240 (9.2)	Reference	0.41
Surgical	37/327 (11.3)	1.26 (0.72–2.20)	
Surgery type of final management§			0.24
Conservative	0/17 (0.0)	—	
Radical	37/310 (11.9)	—	
Time from index TEP to subsequent LMP			
≤ 3 months	4/122 (3.3)	Reference	0.004
3–6 months	13/137 (9.5)	3.09 (0.98–9.75)	
6–18 months	24/199 (12.1)	4.05 (1.37–12.03)	
> 18 months	18/109 (16.5)	5.84 (1.91–17.82)	

Data are given as *n*/*N* (%), unless stated otherwise.

* Odds ratio (OR) given for 5‐year increase in age.

† Prior to index tubal ectopic pregnancy (TEP).

‡ Data available for 55 cases of ectopic pregnancy and 503 total subsequent pregnancies.

§ *P*‐value calculated using Fisher's exact test; unable to calculate OR owing to no ectopic pregnancies in patients following conservative surgery. Embryo, gestational sac containing embryo but no visible cardiac activity; Embryo + CA, gestational sac containing embryo, with visible cardiac activity; GS, empty gestational sac; GS + YS, gestational sac containing yolk sac but no visible embryo; IUCD, intrauterine contraceptive device; LMP, last menstrual period; TOP, termination of pregnancy.

There was no statistically significant difference in the odds of recurrence between expectant management and surgical management of the index TEP (either initial or final management).

The odds of recurrence were more than three times higher in women with two or more previous TEPs compared to women with one previous TEP (i.e. the index TEP only) (OR, 3.27 (95% CI, 1.13–9.42)). Of 19 women in the study who presented with a subsequent pregnancy after two or more TEPs, six (31.6%) did so after successful expectant management of the index TEP, of whom 2/6 (33.3%) had a recurrence of ectopic pregnancy compared with 3/13 (23.1%) of the women who had surgical management.

Women with a history of previous termination of pregnancy (TOP) had lower odds of recurrence compared to those without (OR, 0.45 (95% CI, 0.20–1.03)); however, this comparison did not reach statistical significance (*P* = 0.06). There was no significant difference in the odds of recurrence according to previous miscarriage, parity or previous Cesarean delivery. However, women with ultrasound morphology of the index TEP that demonstrated an embryo with cardiac activity had higher odds of recurrence compared to those with a solid swelling (OR, 2.45 (95% CI, 1.01–5.93)).

Multivariate analysis of the factors was performed with a backwards selection procedure, retaining only the variables with a *P*‐value of < 0.1 in the final model. Table 2 shows the results of this analysis using the retained variables, namely, previous TEP, previous TOP and interpregnancy interval.

**Table 2:** Multivariate analysis of factors associated with odds of recurrent extrauterine ectopic pregnancy (*n* = 567)

Variable	OR (95% CI)	*P*
Previous TOP*		
No	Reference	0.04
Yes	0.40 (0.17–0.94)	
Previous TEP*		
No	Reference	0.02
Yes	3.65 (1.20–11.1)	
Time from index TEP to subsequent LMP		
≤ 3 months	Reference	0.03
3–6 months	2.82 (0.89–8.93)	
6–18 months	3.74 (1.26–11.1)	
> 18 months	5.48 (1.78–16.8)	

* Prior to index tubal ectopic pregnancy (TEP). LMP, last menstrual period; OR, odds ratio; TOP, termination of pregnancy.

### Subgroup analysis of impact of interpregnancy interval according to final management

Analysis of the impact of the interpregnancy interval according to whether the patient had expectant or surgical final management of the index TEP can be seen in Table 3. The odds of recurrence increased significantly with an interpregnancy interval of over 6 months in the group that had expectant management of the index TEP, and over 18 months in the group that had surgical management of the index TEP.

**Table 3:** Recurrence rates following tubal ectopic pregnancy (TEP), according to interpregnancy interval and whether final management of the index TEP was expectant or surgical (*n* = 567)

Final management of index TEP	Subsequent ectopic pregnancy	OR (95% CI)	*P*
Expectant			
Interpregnancy interval			
≤ 3 months	2/70 (2.9)	Reference	0.04
3–6 months	4/59 (6.8)	2.47 (0.43–14.0)	
6–18 months	10/77 (13.0)	5.07 (1.07–24.0)	
> 18 months	6/34 (17.6)	7.29 (1.39–38.3)	
Surgical			
Interpregnancy interval			
≤ 3 months	2/52 (3.8)	Reference	0.15
3–6 months	9/78 (11.5)	3.26 (0.68–15.7)	
6–18 months	14/122 (11.5)	3.24 (0.71–14.8)	
> 18 months	12/75 (16.0)	4.76 (1.02–22.3)	

Data are given as *n*/*N* (%), unless stated otherwise. OR, odds ratio.

### Recurrence after surgical management

Of the 567 women who presented with a subsequent pregnancy, 327 had surgical management as final management of the index TEP. Thirty‐seven (11.9%) of the 310 women who had a salpingectomy had recurrence in their subsequent pregnancy, compared with 0/17 (0%) of those who had conservative surgery (Table 1).

Most recurrences of ectopic pregnancy after salpingectomy were in the contralateral tube (33/37 (89.2%)), but three (8.3%) were in the proximal section of the ipsilateral tube (two in the interstitial portion and one in the tubal stump), and one (2.7%) was within the ipsilateral ovary.

### Recurrence after expectant management

Most women with recurrence of ectopic pregnancy following expectant management of their index TEP had a recurrence in the ipsilateral tube (17/22 (77.3%)).

## DISCUSSION

This study has shown that the odds of recurrent extrauterine ectopic pregnancy was higher in women with a longer interpregnancy interval. Only 3.3% of women with an interval of ≤ 3 months had recurrence of ectopic pregnancy, compared with 16.5% of women with more than 18 months between pregnancies. The odds of recurrence were similar following expectant or surgical management of the index TEP.

Any form of tubal injury would be expected to impede fertilization, delay conception and increase the chance of recurrence. Given that physical resolution of tubal injury after expectant management may take time, it is notable that the lowest rates of recurrence were found in cases in which subsequent conception occurred within 3 months. We reported previously that resolution of TEP on ultrasound imaging generally occurs quickly after normalization of hCG levels^13^ and the data of the present study provide reassurance that tubal function is also restored without delay. An alternative hypothesis could be that significant blockage promotes preferential transport through the contralateral tube, in which a repeat ectopic pregnancy is less likely. Removal of one Fallopian tube, in which damage has caused, or resulted from, TEP, is considered to decrease the risk of recurrence. This has been evidenced previously when comparing salpingotomy with salpingectomy^19^, but this decreased risk may also be assumed when comparing expectant management with salpingectomy. In contrast, our findings demonstrate that expectant management was associated with similar – or, if anything, slightly lower – odds of recurrence. This is consistent with previous literature^20^ and indicates that, alongside being a safe, acceptable and lower‐cost alternative to surgical management^21^, expectant management is not associated with increased odds of recurrence of ectopic pregnancy, and we can reassure women of this. Salient to the interpretation of this result is that management options are dictated predominantly by features of the TEP (such as ultrasound findings, hCG levels or patient symptoms), with those amenable to expectant management generally being physically smaller and on a trajectory to spontaneous resolution.

The data obtained in this study indicate a reassuringly low recurrence rate of ectopic pregnancy after conservative surgery (0/17 (0%)). However, the infrequency of conservative surgery may indicate its selective use on physically smaller, failing TEPs by experienced surgeons; therefore, this low recurrence rate should not be extrapolated further, and results from an existing randomized controlled trial provide stronger evidence to guide decision making^22^.

Smaller and less morphologically advanced TEPs may be expected to cause minimal tubal trauma and could represent failing pregnancies, rather than being caused by tubal blockage. However, as most recurrences in the expectantly managed group were in the ipsilateral tube, salpingectomy may still have conferred a lower risk of recurrence. This may be more important in those with a history of more than one TEP; a small study suggested that such patients have higher rates of further recurrence if treated expectantly, compared with medical or surgical management^23^.

Women with a history of two or more TEPs were found to have three times the odds of ectopic pregnancy recurrence, compared to those with a history of a single TEP. The rate of recurrent extrauterine ectopic pregnancy in this subgroup of patients was 26%, which is consistent with the existing literature^4^, ^5^, ^6^.

Prior TOP is a recognized risk factor for TEP, presumably owing to an association with pelvic infection and emergency contraception use (both of which are recognized as independent risk factors)^6^, ^24^, ^25^. The study of de Bennetot *et al*.^6^ also demonstrated an increased risk of recurrent TEP in women with a history of TOP. However, interestingly, the present study reported that women with a previous TOP had half the odds of recurrence compared to those with no history of TOP, which may reflect evidence of previous successful embryo transport.

In this study, higher odds of recurrence are reported in women for whom embryonic heart pulsations were present in their index TEP. This observation may support a tentative hypothesis that TEP with a live embryo reflects a chromosomally normal pregnancy in a damaged Fallopian tube, rather than a genetically or structurally abnormal pregnancy. This is therefore more likely to be associated with contralateral tubal abnormalities and thus a higher risk of recurrence of TEP in future pregnancies. A more conclusive exploration of this hypothesis would require larger populations in each morphological subgroup.

Despite the large sample size, including patients seen in a busy tertiary hospital over a 12‐year period, the overall number of ectopic pregnancy recurrences was relatively small (*n* = 59). It is possible that a study with a larger sample size may demonstrate further predictors of recurrence. To elucidate the direct impact of management strategy, a randomized controlled trial would be needed, in which women eligible for expectant management would be randomized to either expectant or surgical management. Such a study would be difficult to carry out, in view of the large number of participants required, and a high proportion of patients wishing to exert a preference. It would also be ethically questionable, given the demonstrated efficacy and low recurrence rate following expectant management.

In our analysis, we consistently grouped resolved PULs with correctly sited pregnancies. A proportion of these cases of resolved PUL, especially given their history of TEP, may be cases of resolved TEP. The availability and standard of scanning within our unit minimizes the number of unidentified cases of resolved TEP. Moreover, resolved PULs, by definition, require no physical intervention; therefore, their prediction may be considered to be of less importance.

Regardless of the underlying mechanism, the findings of the present study suggest that women can be reassured about the safety of conceiving shortly after a TEP. This is important, as an unnecessarily cautious approach, based on the theory that functional recovery may take time, may have an adverse impact due to an age‐related decline in fertility.

Future studies could assess the recurrence risk of ectopic pregnancy based on ultrasound or laparoscopic features of concomitant endometriosis, chronic pelvic inflammatory disease or adhesions, which may provide insights into the pathological mechanisms underlying recurrence. It would also be valuable to further examine pregnancies after two (or more) TEPs, to evaluate when surgery should be considered (even if eligible for expectant management) to lower the risk of recurrence.

In conclusion, our data support relaxing any recommendation to delay attempts to conceive after expectant management of TEP. Those with delayed conception and a history of TEP should be considered at particularly high risk of recurrence. Patients can be reassured that expectant management is not associated with higher odds of ectopic pregnancy recurrence.

## Supporting information


**Table S1** Demographic data of women included in study (*n* = 1386)

**Table S2** Ultrasound findings and management outcomes at the time of index tubal ectopic pregnancy diagnosis (*n* = 1386)

**Table S3** Location and management of recurrent extrauterine ectopic pregnancy (*n* = 59)

## Data Availability

The data that support the findings of this study are available on request from the corresponding author. The data are not publicly available due to privacy or ethical restrictions.
