# Supplementary material for: Interpregnancy interval and risk of recurrence following tubal ectopic pregnancy: retrospective cohort study from UK tertiary center
Source: Ultrasound Obstet Gynecol. 2025 Jun 5;66(1):89–95. doi: 10.1002/uog.29262 (PMC12209686; doi:10.1002/uog.29262)
Supplement: Supplementary file 1 — Table S1 Demographic data of women included in study (n = 1386) [file UOG-66-89-s003.docx]

**Supplementary Table 1:** Demographic data of women included in the study (*n=1386)*

| Characteristic |  |
| --- | --- |
| Age (years)  Gravidity  Parity | 31.4 (SD 5.87)  2 (1-12)  0 (0-7) |
| Previous miscarriage  Previous termination of pregnancy  Previous Caesarean section  Previous tubal ectopic pregnancy | 343 (24.7)  328 (23.7)  171 (12.3)  59 (4.3) |
| Intrauterine contraceptive device in situ | 25 (1.8) |

*Data are given as mean (Standard Deviation), median (range) or n (%)*
