# Supplementary material for: Interpregnancy interval and risk of recurrence following tubal ectopic pregnancy: retrospective cohort study from UK tertiary center
Source: Ultrasound Obstet Gynecol. 2025 Jun 5;66(1):89–95. doi: 10.1002/uog.29262 (PMC12209686; doi:10.1002/uog.29262)
Supplement: Supplementary file 2 — Table S2 Ultrasound findings and management outcomes at the time of index tubal ectopic pregnancy diagnosis (n = 1386) [file UOG-66-89-s001.docx]

**Supplementary Table 2:** Ultrasound findings and management outcomes at the time of index tubal ectopic pregnancy diagnosis (n=1386)

|  |  | n (%) |
| --- | --- | --- |
| **Morphology** | Solid swelling  Gestational sac  Yolk sac  Embryo  Embryo with cardiac activity | 609 (43.9)  488 (35.2)  110 (7.9)  40 (2.9)  139 (10.0) |
| **Initial management** | Expectant  Surgical | 739 (53.3)  647 (46.7) |
| **Final management** | Expectant  Surgical | 504 (36.4)  882 (63.6) |
