# Supplementary material for: Interpregnancy interval and risk of recurrence following tubal ectopic pregnancy: retrospective cohort study from UK tertiary center
Source: Ultrasound Obstet Gynecol. 2025 Jun 5;66(1):89–95. doi: 10.1002/uog.29262 (PMC12209686; doi:10.1002/uog.29262)
Supplement: Supplementary file 3 — Table S3 Location and management of recurrent extrauterine ectopic pregnancy (n = 59) [file UOG-66-89-s002.docx]

**Supplementary Table 3:** Location and management of recurrent extrauterine ectopic pregnancy (n=59)

|  |  | n (%) |
| --- | --- | --- |
| Location | Tubal (contralateral)  Tubal (ipsilateral) Ovarian | 37 (62.7)  21 (35.6)  1 (1.7) |
| Morphology | Solid swelling Gestational sac Yolk sac Embryo Embryo with cardiac activity | 23 (39.0) 23 (39.0) 3 (5.1) 2 (3.4) 8 (13.6) |
| Initial management of recurrent ectopic pregnancy | Expectant  Medical  Surgical | 36 (61.0)  0 (0.0)  23 (39.0) |
| Final management of recurrent ectopic pregnancy | Expectant  Medical  Surgical | 27 (45.8)  1 (1.7)  31 (52.5) |
